# Predictors of pregnancy among young people in sub-Saharan Africa: a systematic review and narrative synthesis

**DOI:** 10.1136/bmjgh-2019-001499

**Published:** 2019-06-05

**Authors:** Nathali Gunawardena, Arone Wondwossen Fantaye, Sanni Yaya

**Affiliations:** 1 Faculty of Science, University of Ottawa, Ottawa, Ontario, Canada; 2 Faculty of Health Sciences, University of Ottawa, Ottawa, Ontario, Canada; 3 School of International Development and Global Studies, University of Ottawa, Ottawa, Ontario, Canada

**Keywords:** teenage pregnancy, predictors, sub-saharan africa, systematic review

## Abstract

**Background:**

Sub-Saharan Africa has among the highest prevalence of teenage pregnancy in the world. Teenage mothers and their children are at risk to a host of medical, social and economic challenges. Adolescent pregnancy is a significant cost to the mother and newborn child, and also to their family and the wider society. Despite measures taken by some sub-Saharan nations to tackle the issue of adolescent pregnancy, the phenomenon remains a public health concern that is widespread throughout the region. Currently, there are few studies that examine the predictors of teenage pregnancy in the sub-Saharan region. The objective of the present study was to systematically review predictors of pregnancy among young people in sub-Saharan Africa.

**Methods:**

A literature search was conducted using Medline, CINAHL and EMBASE electronic databases. Following duplicate removal, abstract and full-text screening, 15 studies were ultimately included in the final review. Narrative synthesis was used to synthesise the qualitative and quantitative findings. This review followed the Preferred Reporting Items for Systematic Reviews and Meta-analyses.

**Results:**

Twenty-seven predictors of teenage pregnancy were identified and grouped into six themes (Partner and peer-related predictors; Sexual health knowledge, attitude and behaviour-related predictors; Parenting and family-related predictors; Economic, environmental and cultural predictors; Personal predictors; and Quality of healthcare services predictors). The most obvious predictors included sexual coercion and pressure from male partners, low or incorrect use of contraceptives, and poor parenting or low parental communication and support.

**Conclusion:**

This review emphasises that the large prevalence of adolescent pregnancy in sub-Saharan Africa is attributable to multiple predictors that our study was able to group into six themes. Policy changes and programmes must be implemented in sub-Saharan Africa to address these determinants in order to reduce adolescent pregnancy within the region.

**Key questions**

**What is already known?**

- Teenage pregnancy constitutes a large public health concern in sub-Saharan Africa that is associated with a host of complications for mother and child.
- Currently, there are relatively few studies that have examined predictors of pregnancy among young people in the region.

**What are the new findings?**

- Sexual coercion or pressure from male partners, low or incorrect use of contraceptives, lack of parental communication and support, and poor parenting are common predictors of teenage pregnancy.
- There is currently a gap in research regarding the predictors associated with teenage pregnancy within the countries with the highest rates in sub-Saharan Africa.

**What do the new findings imply?**

- Policy-makers should focus on changing attitudes involving sexual coercion and violence at the community and interpersonal level, encouraging community discussions that dispel myths about contraceptive use, encouraging the involvement of parents in pregnancy prevention programmes, improving comprehensive sexual education programmes, increasing adolescent-friendly health services in facilities, alleviating poverty, increasing employment, increasing economic growth and lowering school dropout.
- Further research is needed to identify predictors of teenage pregnancy in other sub-Saharan African countries, especially countries with the highest prevalence of the phenomenon.

## Background

Throughout the world, in both developed and developing nations, teenage pregnancy constitutes a public health concern.^1–3^ A finding by Save the Children organisation suggested that worldwide, 13 million children are born to women under 20 years of age, with 90% of these births taking place in developing countries.^4^ In developing nations, complications resulting from pregnancy and childbirth are the leading cause of maternal mortality in young women aged 15–19, with the highest risk being in Africa, Afghanistan, Bangladesh, Guatemala, Haiti, Nepal, Nicaragua and Yemen.^5^ The risk of death due to pregnancy-related complications is double in adolescents compared with women who are in their twenties.^6^ Adolescent women are at risk of unwanted pregnancy, and they are also susceptible to sexually transmitted infections as well as coerced sexual relationships.^7^

Adolescent mothers are also more likely to take part in unsafe abortions and become young mothers for a second time.^8–10^ In addition, teenage mothers often have poor future prospects including school dropout.^11^ Few job opportunities exist for young women who drop out of school due to pregnancy. This can result in a cycle of poverty in these women’s families. Adolescent mothers are at risk, and so are their offspring. Infants who are born to adolescent mothers are more likely to be born premature and face a higher risk of dying compared with infants born to older mothers who are between the ages of 20 and 24.^8 9 12 13^ These children often face a host of problems including poor development, malnutrition and inadequate education.^9 14^

In 2013, sub-Saharan Africa had the highest prevalence of adolescent pregnancy in the world.^15^ Half of all births that occurred in the region were to teenage mothers, with an estimated 101 births per 1000 women aged 15–19, almost double the global average.^15 16^ Out of the 15 countries in the world that had more than 30% of young women 20–24 years old giving birth before the age of 18, 14 were in sub-Saharan Africa.^17^ Some of these countries include Cameroon, Malawi, Mozambique, Niger, and Uganda.

Several sub-Saharan African nations have taken measures to tackle to the issue of adolescent pregnancy. Slight reduction of teenage pregnancy and school dropout levels were shown in Kenya after the introduction of programmes training teachers on HIV and providing girls with education subsidies.^18^ In Cameroon, programmes that incorporated peer education to educate girls on disease, pregnancy, sexuality, peer pressure and dating were used to empower young women to make the right choices.^19^ In Madagascar, youth-friendly clinics were introduced in 2001.^19^ These clinics provided low-cost and confidential access to contraceptives, counselling and diagnosis of sexually transmitted infections. Despite such measures taken by several governments to reduce the phenomenon of adolescent pregnancy in their countries, girls falling pregnant during their teenage years continues to be a cause of concern in sub-Saharan Africa.

Despite the high prevalence of adolescent pregnancy in sub-Saharan Africa and the host of negative medical, social and economic consequences that are associated with the problem, relatively few studies have examined predictors of pregnancy among young people in the region. The following study aims to systematically review the predictors of teenage pregnancy in sub-Saharan Africa. Identification and understanding of these predictors can help policy-makers design and implement interventional programmes to reduce adolescent pregnancy in the region.

## Methods

### Search strategy

A systematic review of English literature was performed on 7 October 2018 to identify primary research articles focusing on predictors of teenage pregnancy in sub-Saharan Africa. The articles were published from 2008 to 2018. This time frame was chosen in order to limit the study to contemporary studies, as the predictors of teenage pregnancy in older studies may no longer be relevant. An electronic search was conducted using online databases MEDLINE, EMBASE, and Cumulative Index to Nursing and Allied Health Literature (CINAHL). The search strategy is shown in online appendix A. This review was developed and reported according to the Preferred Reporting Items for Systematic Reviews and Meta-analyses (PRISMA) 2009 checklist (see online appendix B for PRISMA flow diagram and online appendix E for PRISMA checklist).

10.1136/bmjgh-2019-001499.supp1 Supplementary data

10.1136/bmjgh-2019-001499.supp2 Supplementary data

10.1136/bmjgh-2019-001499.supp5 Supplementary data

### Eligibility criteria

Eligibility for inclusion in the review were primary, original research studies published in English, reporting on predictors of teenage pregnancy in sub-Saharan Africa. Included studies used quantitative, qualitative or mixed-methods approaches. Excluded studies were those not taking place in sub-Saharan Africa or those examining negative health behaviours and outcomes after the pregnancy. Also excluded were conference abstracts, editorials, commentaries, study protocols, news articles and secondary analyses (eg, reviews or meta-analyses). Some studies used non-adolescent key informants: older women who had been pregnant as a teenager or older men who had impregnated a young woman while they were a teenager. These informants were included if they mentioned predictors of pregnancy in the adolescent years specifically.

### Study selection

Abstracts were screened according to the aforementioned criteria, and full texts were retrieved for eligible studies. At full-text review, in addition to the aforementioned criteria, studies were excluded if they did not focus on predictors of pregnancy specifically in adolescents (age 13–19 years). Two investigators independently screened the titles and abstracts of articles retrieved from the literature search, and the full texts of potentially eligible articles were obtained and further assessed for final inclusion. Disagreements were resolved through consensus. When a consensus could not be reached, a third reviewer was used come to a final decision.

### Data collection and risk of bias assessment

Zotero was used for de-duplication and screening (Zotero software; Center for History and New Media, George Mason University). Data extraction was carried out in Microsoft Excel (V.15.0.5101.1002). The authors assessed the reporting of included qualitative studies using the criteria based on the Critical Appraisal Skills Programme’s (CASP) 10 questions for qualitative research^20^ in online supplementary appendix D table 2 . CASP was selected due to its extensive previous use for systematic reviews of qualitative studies. The domains of the CASP checklist helped assess the credibility of the qualitative findings and the rigour of the studies.^21^ The 10 questions were designed as prompts to guide reviewers in critically reading the reports. Included studies were assigned an overall score of ‘high’ (9-10), ‘moderate’ (7.5–9) or ‘low’ (less than 7.5) overall quality. The authors assessed the reporting of included quantitative studies using the criteria based in the Quality Assessment Tool for Quantitative Studies by the Effective Public Health Practice Project^22^ in online supplementary appendix D table 3. Each study was assessed based on six domains and given a score of strong, moderate or weak. Then an overall score was given for each study based on the sum of values given on the six domains. Global rating for the studies were determined by strong (no weak ratings), moderate (one weak rating) and weak (two or more weak ratings).

10.1136/bmjgh-2019-001499.supp4 Supplementary data

The authors assessed the reporting of included mixed-methods studies using the criteria based in the Mixed Methods Appraisal Tool^23^ in online supplementary appendix D tables 4-6. Each study was given a score of 1 (yes) or 0 (no) on various domains depending on if they met certain listed criteria. The scores for each domain were then added to give an overall score. Studies were not excluded or weighted based on the quality of the reporting assessments. The results of the appraisals and assessments were instead used to inform data interpretation and ultimately determine trustworthiness of review findings and conclusions.

### Data analysis

An electronic data collection form was developed by the first investigator (NG). On conducting the three levels of screening, two investigators (NG and SY) used the data extraction tool to independently extract the following information from each article: (1) sample size; (2) country where the study was conducted in; (3) study design (quantitative, qualitative and mixed); (4) data collection procedures (self-administered structured questionnaire, focus group, etc); (5) sampling strategy; (6) predictors of adolescent pregnancy. Extracted data can be found in online supplementyary appendix C table 1.

10.1136/bmjgh-2019-001499.supp3 Supplementary data

### Method of synthesis

The method of synthesis chosen for this review was the narrative synthesis method using textual narrative summaries to analyse the data, based on the guidelines by Popay *et al*.^24^ This method is useful for describing and identifying patterns and differences between multiple studies from diverse sources in order to address or examine an issue.^24^ Narrative synthesis allows primary data to be summarised and described in a clear and structured manner allowing gaps in research to be found more easily and future research implications to be provided.^24 25^ In a narrative synthesis, identified factors are combined into more homogenous groups. We grouped the multiple predictors of teenage pregnancy found in our selected studies into six themes (Partner and peer-related predictors; Sexual health knowledge, attitude and behaviour-related predictors; Parenting and family-related predictors; Economic, environmental and cultural predictors; Personal predictors; and Quality of healthcare services predictors). Similar predictors of teenage pregnancy were grouped into the same theme, making it easier to identify patterns between studies.

### Patient and public involvement

Patients and public were not involved in the design and conduct of this research.

## Results

### Search results

The literature search returned 435 studies, with 336 remaining after duplicate removal. Two hundred ninety-five studies were excluded after abstract screening, and thus 41 full articles were screened. Following 26 exclusions at full-text level (mainly due to only reporting about negative health behaviours and outcomes after pregnancy), 15 studies were included in the final review. Online appendix B shows the study screening and inclusion process.

### Study characteristics

Articles included in this review were studies conducted in sub-Saharan African countries with a focus on adolescent pregnancies. Of the 15 included studies, 5 were conducted in South Africa, 3 in Ghana, 2 in Ethiopia, 2 in Tanzania, 2 in Nigeria and 1 in Malawi. Eight studies used quantitative research methods, five used qualitative research methods and two used a mixed-methods approach.

Most of the participants included in the study were adolescents. Studies were conducted in rural, urban and semi-urban settings. The number of participants per study varied from 19 to 3123. The total approximate number of participants was 8087. Refer to online supplementyary appendix C table 1 for detailed descriptions of participant characteristics. At least 228 key informants comprising health workers, community members, community leaders, teachers and parents were involved in some of the studies. These key informants provided information regarding some of the predictors of teenage pregnancy. Online supplementyary appendix C table 1 provides further details on study characteristics.

### Partner and peer-related predictors

Sexual coercion or pressure from male partners was cited as a predictor of teenage pregnancy in 6 of the 15 studies.^26–31^ In one study conducted in South Africa, pressure from one’s partner to not use contraceptives or to have a baby was stated by numerous young women as the reason why they became pregnant as an adolescent.^28^ One girl in this study stated that her boyfriend insisted on having a baby before marriage, so she complied. In another study, teenage girls engaged in sexual relations with men in exchange for gifts and money or due to relentless pressure from the man to have sex.^29^ Peer influence and the need to maintain one’s social status among peers was cited as a predictor in four of the studies.^26 27 30 31^ In one study, teenage mothers cited trying to fit in with friends as a reason for engaging in sexual activity.^26^ In a Nigerian study, girls cited pressure from peers to get married as a contributor to teenage pregnancy.^31^

### Sexual health knowledge, attitude and behaviour-related predictors

Low or incorrect use of contraceptives was cited in 6 out of the 15 studies.^27 28 30 32–34^ In one study conducted in Malawi, misconceptions about contraceptives due to lack of knowledge and low awareness led to low contraceptive use.^32^ Misconceptions included sterility, condoms disappearing within the woman’s body, development of cancer, prolonged menstruation, heart palpitations, and excessive weight gain or loss. In another study, girls avoided using contraceptives simply because they did not want to use them.^27^ Low knowledge of sexual health, contraceptives and abortion was mentioned in 4 of the 15 studies.^27 28 30 33^ In one study conducted in South Africa, more than half of female respondents (74.1%) fell pregnant due to lack of knowledge about pregnancy.^33^ In another study, some adolescent girls did not even know how pregnancy occurs.^28^ Finally, fear of abortion and using birth control was cited as a predictor in one study.^28^ In this study, one participant stated her fear of using needles as a leading factor for non-use of contraceptive injections.

### Parenting and family-related predictors

Lack of parental communication and support, or poor parenting were cited as predictors, being reported in five of the studies.^26 34–37^ In a study from Ghana, girls cited parents not being present in the household as a reason why they were susceptible to involvement in sexual relationships with men.^26^ In another study from Ghana, 74.6% of girls who were highly resilient to pregnancy cited their parents as a source of social support compared with 38.6% of low-resilience girls.^36^ Family instability, breakdown and parental divorce were cited as predictors in three of the studies.^30 31 38^ In a study in Ethiopia, respondents whose parents lived together had lesser odds of having sexual activities than those with divorced or separated parents.^38^ History of early sexual activity or marriage in the family was cited as a predictor in two studies.^35 38^ In northern Ethiopia, a history of maternal teenage pregnancy represented a predictor of teenage pregnancy.^35^ Having parents living in rural areas was cited as a predictor in one study.^38^ Having been born out of wedlock was cited as a predictor in one study.^38^

### Economic, environmental and cultural predictors

Low socioeconomic status, economic constraints, low income and a lack of employment opportunities were cited as predictors in 4 out of the 15 studies.^26 32 33 35^ In a South African study, lack of employment and job opportunities was found to be associated with teenage pregnancy.^33^ Sexual abuse and rape were cited as a predictor in three of the studies.^29 31 32^ In a Tanzanian study, girls reported rape as a cause for concern.^29^

Cultural practices and early marriage were also cited as predictor in 3 out of the 15 studies.^32 35 39^ In one study, significantly more married (93.3%) than unmarried (41.9%) teenagers had been involved in pregnancy.^39^ Poverty was cited as a predictor in two studies.^29 30^ One study suggested that girls living in poverty engaged in sexual relations with older men in exchange for money in order to meet their basic needs or support their family.^29^ Exposure to indecent and sex-based messages in the media was cited as a predictor in two studies.^26 30^ Both male and female teenagers in rural Ghana expressed wanting to try sexual activities exhibited in scenes from movies or television.^26^ Lack of religious commitment was cited as a predictor in one study.^31^ Finally, having low access to cultural capital (mass media) was cited as a predictor in one study.^40^

### Personal predictors

Low educational expectations and school dropout were mentioned in 3 out of the 15 studies.^30 32 39^ In one study conducted in Ghana, more out-of-school (68.1%) than in-school (18.8%) female teenagers had ever been pregnant.^39^ Low self-esteem or wanting to prove one’s identity were cited as predictors in two of the studies.^30 33^ In one study, 19% of respondents got pregnant because they wanted to prove their identity and maturity as a woman.^33^ Curiosity was cited as a predictor in two of the studies.^26 27^

Ignorance was cited as a predictor in two of the studies.^27 31^ In a Nigerian study, all groups of teens interviewed indicated that their peers believed that they were invulnerable to pregnancy.^27^ Older age (being in one’s late teens) was cited as a predictor in one of the studies.^32^ Finally, deliberately choosing to have a child or to not follow information provided in sexual education classes were cited as predictors in one of the studies.^28^ A girl in this study describes how she deliberately ignored sexual education classes saying “I was not listening in class because I don’t like topics on pregnancy prevention, and Life Orientation class is the one I was not attending, I was going out when it starts” (p.5).

### Quality of healthcare services predictors

Non-friendly adolescent reproductive services, lack of sexual health knowledge provided by healthcare workers and negative attitudes of health workers towards providing reproductive health services for adolescents were mentioned as predictors in three of the studies.^27 30 34^ In a Nigerian study, some girls reported poor treatment by healthcare workers.^27^ This poor treatment included being shouted at and receiving nasty remarks. In South Africa, girls reported that nurses would deliberately insult girls seeking contraceptives, withhold information on the correct use of contraceptives and would tell the girls that they are too young to be sexually active.^30^ One girl in this same study describes her experience stating “when I went to get contraceptives, the nurses told me that I am a loose woman and I will become pregnant. But I was not. I was just innocent. They did not tell me straight what I must do or what I must not do”.

## Discussion

The current study identified predictors of adolescent pregnancy in sub-Saharan Africa. Fifteen articles that examine predictors of teenage pregnancy in sub-Saharan Africa were found by systematically searching three electronic databases. The most commonly cited predictors of teenage pregnancy included sexual coercion or pressure from male partners, low or incorrect use of contraceptives, lack of parental communication and support, or poor parenting.

Sexual coercion or pressure from male partners was one of the most commonly cited predictors of teenage pregnancy. Sexual coercion is where a person is forced to have sex against their will with the use of violence, threats, deception or economic circumstances.^41^ In sub-Saharan Africa, research shows that 15%–68% of young people encountered at least one experience of sexual coercion.^42–46^ Young women who have been sexually coerced tend to have a higher prevalence of unwanted pregnancy.^47^ The association between sexual coercion and teenage pregnancy can be explained by a variety of reasons. First, pregnancy may occur as a direct result of forced sex.^47^ Second, victims often have little power to negotiate contraceptive use.^48^ Finally, young women who have experienced sexual coercion tend to engage in risky sexual behaviours.^42 49^ A study by Sathyanarayana *et al* suggests that a restructuring of social institutions and parenting to analyse the processes involved in sexual violence is required in order to lower the prevalence of sexual coercion.^50^

Low or incorrect use of contraceptives was the other most commonly cited predictor of teenage pregnancy. Many girls had misconceptions about contraceptives resulting in them choosing not to use available methods. In a study of eight developing countries, 50%–70% of women believed that they would face considerable health challenges if they used the pill.^51^ Another study in Kenya found that many young sexually active women had misconceptions about the side effects of contraceptives.^52^ These misconceptions can spread in social networks leading to community-wide negative perceptions about contraceptives.^53 54^ In Nigeria, fear of the side effects of contraceptive use was a major cause for non-use.^55^ Programmes encouraging community discussions of contraceptives are needed to dispel myths and misconceptions.^56^

The next most commonly cited predictor of teenage pregnancy was lack of parental communication and support, or poor parenting. Numerous studies have linked parental support and connectedness with lower frequency of teen intercourse, lower sexual risk taking and delayed sexual debut.^57–59^ Improved parent connectedness, communication and increased involvement of parents in pregnancy prevention programme could lower the prevalence of teenage pregnancy and sexual activity.^60^

Another commonly cited predictor of teenage pregnancy was low socioeconomic status, economic constraints, low income or lack of employment opportunities. Several previous studies have found low family income to be a predictor of teenage pregnancy.^61–64^ One possible reason for this relationship may be that teenage girls with higher incomes may continue in their education and pursue higher career goals, while teenage girls from low-income families may settle for an early marriage.^65–67^ Prior studies in Ghana, South Africa and Tanzania have indicated that low socioeconomic status and economic constraints can lead adolescent girls to pursue sexual relationships with older men in order to meet their financial needs.^68–70^ Another South African study found that some adolescents get pregnant intentionally in order to receive financial support from the government.^71^ Policies that ensure poverty alleviation, higher levels of adult education, and employability are needed to simultaneously increase economic growth and decrease teenage pregnancy.^72^

Low educational expectations and school dropout were also commonly associated with teenage pregnancy. Being out of school can lead to risky sexual behaviour, pregnancy and early marriage.^73^ In a Kenyan study, women with a secondary school education had their first sexual intercourse 3 years later than women with no education.^74^ In South Africa, falling behind in school was the strongest predictor of becoming pregnant within the following 2 years.^75^ In a review looking at the determinants of adolescent sexual health in developing nations, high school performance and motivation to continue one’s education served as protective factors.^76^ Out-of-school teenagers are more likely to have sex as well as have multiple life partners.^77^ Adolescents who stay in school and perform well in their studies may have a stronger perception of the risks that are associated with early sexual activity and may also have higher aspirations for their future that deter them from engaging in sex.^78 79^ Teaching-related interventions can possibly improve school progression and learning for some students^80–82^; however, other factors like poverty, peers, family and community pressures have also been associated with school dropout.^83–86^ These factors may require larger, more complex solutions.

Through a comprehensive electronic search, our study has revealed a multitude predictors of pregnancy among young people in sub-Saharan Africa. There are relatively few studies that have been conducted in relation to teenage pregnancy in sub-Saharan Africa, meaning that our study can help fill gaps in research.

This systematic review has several different limitations. The first limitation is that data sources for the study were published in three online databases including Medline, CINAHL and EMBASE. Only studies published in the English language were used for the review. Due to these reasons, there is a chance that other relevant studies describing predictors of teenage pregnancy may have been missed for the reason that they may have been published elsewhere or in a language other than English.

Studies included in this review were conducted in specific sub-Saharan African countries (South Africa, Ghana, Ethiopia, Tanzania, Nigeria and Malawi). Although it is likely that the same predictors found in this review exist in other sub-Saharan African nations, additional predictors associated with teenage pregnancy may exist in these other countries. Interestingly, only one of the countries included in our study, Malawi, makes up the top seven countries in sub-Saharan Africa with the highest teenage pregnancy rates.^87^ This indicates a gap in research regarding the predictors associated with teenage pregnancy within the countries with the highest rates.

Grey literature, reports and other non-peer reviewed studies were not included in this review, adding to the limitations. Finally, predictors identified from the articles are difficult to compare as a wide range of sampling strategies, data collection methods and study designs were used in the different papers.

## Conclusion

Many predictors of adolescent pregnancy in sub-Saharan Africa exist that result in countless unwanted pregnancies and a range of consequences for young mothers, their children and the wider society. Policy-makers should focus on changing attitudes involving sexual coercion and violence at the community and interpersonal level, encouraging community discussions that dispel myths about contraceptive use, encouraging the involvement of parents in pregnancy prevention programmes, improving comprehensive sexual education programmes, increasing adolescent friendly health services in facilities, alleviating poverty, increasing employment opportunities, increasing economic growth and lowering school dropout. Further research is needed to identify predictors of teenage pregnancy in other sub-Saharan African countries, especially countries with the highest prevalence of the phenomenon.
