# Supplementary material for: Predictors of pregnancy among young people in sub-Saharan Africa: a systematic review and narrative synthesis
Source: BMJ Glob Health. 2019 Jun 5;4(3):e001499. doi: 10.1136/bmjgh-2019-001499 (PMC6570986; doi:10.1136/bmjgh-2019-001499)
Supplement: Supplementary data [file bmjgh-2019-001499supp001.pdf]

Search Strategy Example (Embase)

1. “africa south of the sahara"/ or angola/ or benin/ or botswana/ or burkina faso/ or burundi/ or cameroon/ or cape verde/ or central africa/ or central african republic/ or chad/ or comoros/ or congo/ or cote d'ivoire/ or democratic republic congo/ or djibouti/ or equatorial guinea/ or eritrea/ or ethiopia/ or gabon/ or gambia/ or ghana/ or guinea/ or guinea-bissau/ or kenya/ or lesotho/ or liberia/ or madagascar/ or malawi/ or mali/ or mayotte/ or mozambique/ or namibia/ or niger/ or nigeria/ or rwanda/ or sahel/ or senegal/ or sierra leone/ or somalia/ or south africa/ or south sudan/ or sudan/ or swaziland/ or tanzania/ or togo/ or uganda/ or zambia/ or zimbabwe/
2. adolescent/
3. teen.mp.
4. young people.mp.
5. 2 or 3 or 4
6. predictors.mp.
7. factors.mp.
8. determinants.mp.
9. reasons.mp.
10. influences.mp.
11. sociocultural factors.mp.
12. 6 or 7 or 8 or 9 or 10 or 11
13. adolescent pregnancy/
14. adolescent pregnancy.mp.
15. unintended pregnancy.mp. or unplanned pregnancy/
16. teenage pregnancy.mp.
17. teen pregnancy.mp.
18. 13 or 14 or 15 or 16 or 17
19. 1 and 5 and 12 and 18
20. limit 19 to yr="2008 - 2018"
