# Supplementary figures and images for: Predictors of pregnancy among young people in sub-Saharan Africa: a systematic review and narrative synthesis
Source: BMJ Glob Health. 2019 Jun 5;4(3):e001499. doi: 10.1136/bmjgh-2019-001499 (PMC6570986; doi:10.1136/bmjgh-2019-001499)

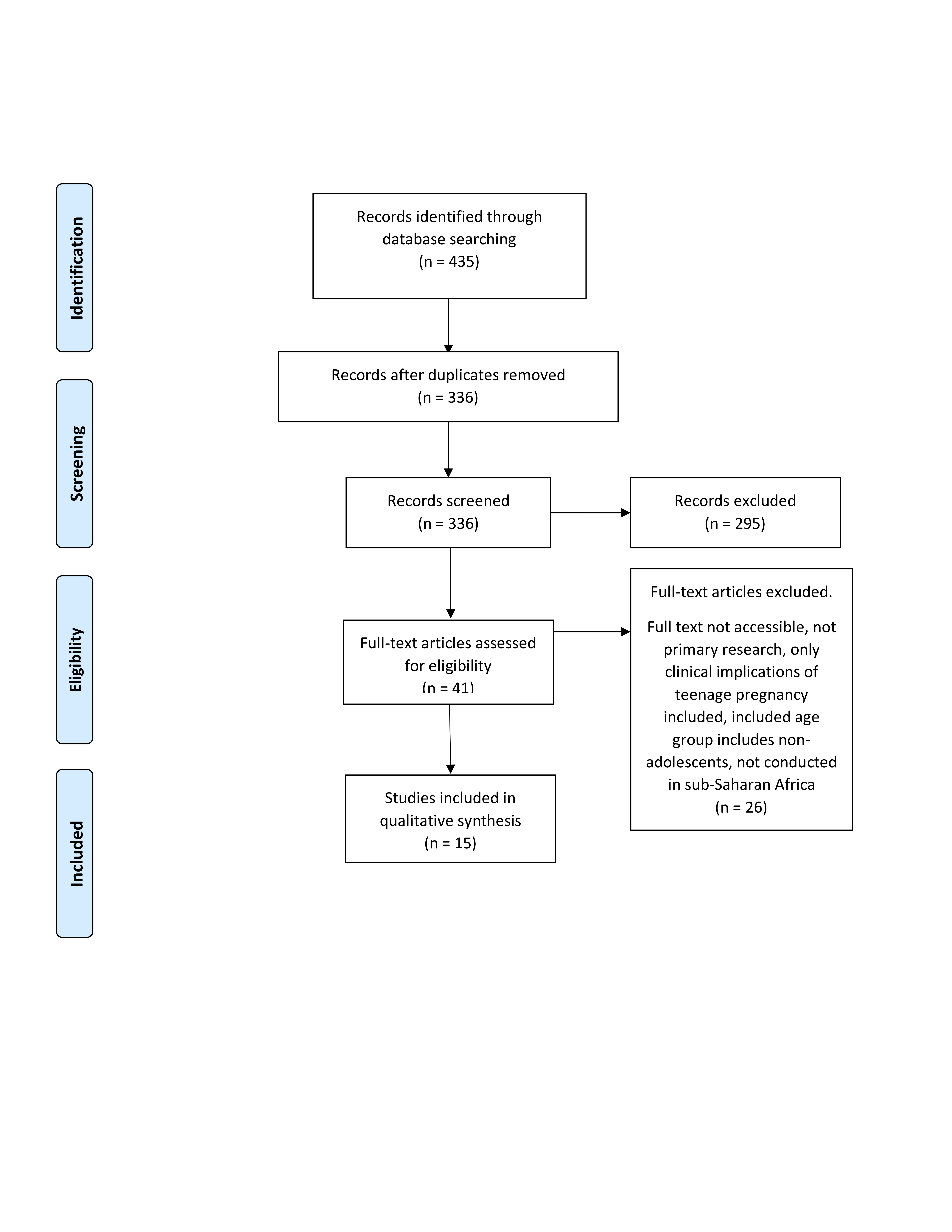

Supplement: Supplementary data [file bmjgh-2019-001499supp002.jpg]
