# Supplementary material for: Predictors of pregnancy among young people in sub-Saharan Africa: a systematic review and narrative synthesis
Source: BMJ Glob Health. 2019 Jun 5;4(3):e001499. doi: 10.1136/bmjgh-2019-001499 (PMC6570986; doi:10.1136/bmjgh-2019-001499)
Supplement: Supplementary data [file bmjgh-2019-001499supp004.pdf]

Appendix D
Quality Appraisals

Table 2. Summary of quality scores for Qualitative studies based on 10 CASP checklist questions

**Qualitative studies**	Adongo et al	Lebese et al	Masemola-Yende & Mataboge	McCleary - Sills et al	Mkhwanazi
Was there a clear statement of research aims	1	1	1	0.5	0.5
Is a qualitative methodology appropriate	1	1	1	1	1
Was the research design appropriate to address the aims of the research	1	1	1	1	0.5
Was the recruitment strategy appropriate to the aims of the research	1	1	1	0.5	0.5
Was the data collected in a way that addressed the research issue	1	1	1	0.5	1
Has the relationship between researcher and participants been adequately considered	0	0	0.5	0.5	0.5
Have ethical issues been taken into consideration	1	1	1	1	1
Was the data analysis sufficiently rigorous?	1	1	0.5	0.5	0.5
Is there a clear statement of findings	1	1	1	1	1
How valuable is the research?	1	1	1	1	1
**Overall Quality**	**9**	**9**	**9**	**7.5**	**7.5**

Table 3. Summary of quality scores based on Effective Public Health Practice Project (EPHPP) Quality Assessment Tool for Quantitative Studies

**Quantitative Studies**	Ayele et al	Kaphagawani & Kalipeni	Mchunu et al	Morhe et al	Mothiba & Maputle	Ochiogu et al	Pfeiffer et al	Salih et al
Selection Bias	strong	strong	moderate	moderate	strong	moderate	strong	strong
Study Design	moderate	moderate	moderate	moderate	moderate	moderate	moderate	moderate
Confounders	strong	weak	weak	moderate	weak	moderate	moderate	weak
Blinding	weak	weak	weak	weak	weak	weak	weak	weak
Data Collection Methods	moderate	moderate	strong	strong	strong	moderate	strong	moderate
Withdrawals and Dropouts	N/A	N/A	N/A	N/A	N/A	moderate	N/A	N/A
**Overall Quality**	**moderate**	**weak**	**weak**	**moderate**	**weak**	**moderate**	**moderate**	**Weak**

Quality Appraisals

Mixed Studies

Table 4. Quality Assessment of the Qualitative Component of Mixed Methods Studies using the Mixed Methods Appraisal Tool (MMAT)

**Qualitative component**	Gyan et al	Kuyinu et al
Source of data	1	1
Methods of analysis	1	1
Context	0	0
Reflexivity	0	0
**Overall**	**2**	**2**

Table 5. Quality Assessment of the Quantitative Descriptive Component of Mixed Method Studies using the Mixed Methods Appraisal Tool (MMAT)

**Quantitative descriptive component**	Gyan et al	Kuyinu et al.
Sampling strategy	1	1
Representative sample	1	1
Appropriate measurement	1	1
Response rate	1	1
**Overall**	**4**	**4**

Table 6. Quality Assessment of Mixed Designs using the Mixed Methods Appraisal Tool (MMAT)

**Mixed Methods Design**	Gyan et al	Kuyinu et al.
Relevant Design	1	1
Integration of Data	1	1
Limitations	0	0
**Overall**	**2**	**2**
