# Supplementary material for: Predictors of pregnancy among young people in sub-Saharan Africa: a systematic review and narrative synthesis
Source: BMJ Glob Health. 2019 Jun 5;4(3):e001499. doi: 10.1136/bmjgh-2019-001499 (PMC6570986; doi:10.1136/bmjgh-2019-001499)
Supplement: Supplementary data [file bmjgh-2019-001499supp003.pdf]

Reference	Sample Size	Location, Setting	Design	Data Collection	Sampling Strategy	Predictors of Pregnancy Among Young People
**Adongo [26]**	21	Ghana, rural	Qualitative	In-depth interviews	Purposive sampling	- Sexual favors in return for tutoring<br>- Social fitness<br>- Poor parenting<br>- Economic constraints<br>- Curiosity<br>- Indecent media content
**Ayele et al. [35]**	414	Ethiopia, rural	Quantitative	Community-based case control study	Random sampling	- marriage<br>- history of maternal teenage marriage in family<br>- lack of communication with parents<br>- lower monthly household income
**Gyan et al. [36]**	419	Ghana, urban	Mixed	Questionnaire, interviews	Multistage cluster sampling, random sampling	- Lack of social support from family
**Kaphagawani & Kalipeni [32]**	505	Malawi, rural and urban	Quantitative	Questionnaire, interviews	Stratified sampling, simple random sampling	- School dropout<br>- Low socio-economic status<br>- Older age (being in one's late teens)<br>- Cultural practices<br>- Forcible rape<br>- Low contraceptive use<br>- misinformation from traditional counsellors about contraceptives and family planning
**Kuyinu et al. [27]**	46	Nigeria, urban	Mixed	Focus group discussions, key informant interviews, data extraction from healthcare facility registries	Purposive sampling	- peer pressure from partner<br>- peer pressure from peers<br>- maintaining social status in one's peer group<br>- curiosity<br>- belief that one is invulnerable to pregnancy<br>- not using contraceptives<br>- little knowledge about contraceptives<br>- lack of youth friendly health services

						- unfriendly health workers
**Lebese et al. [37]**	42	South Africa, rural	Qualitative	Focus group discussions, interviews	Purposive sampling	- communication issues between parents and children regarding sex
**Masemola – Yende & Matabone [28]**	19	South Africa, urban	Qualitative	Semi-structured interviews	Purposive sampling	- pressure from partner to not use contraceptives<br>- chose to have a baby with partner<br>- fear of abortion so chose not to terminate pregnancy<br>- non-constant birth control or condom use<br>- using risky alternative medication along with hormonal contraceptives<br>- fear of using birth control<br>- deliberately choosing not to follow information provided in sexual education classes
**McCleary-Sills et al. [29]**	223	Tanzania, rural	Qualitative	Participatory learning and action (PLA) exercises, focus group discussions, key informant interviews	Sampling method could not be determined	- rape and coercive sex<br>- sexual expectations of men<br>- poverty
**Mchunu et al. [33]**	3,123	South Africa, urban and rural	Quantitative	Cross-sectional population-based household survey	Multi-stage stratified cluster sampling	- lack of employment opportunities<br>- wanted to prove maturity and identity as a woman<br>- low condom use<br>- low sexual-health knowledge
**Mkhwanazi [34]**	40	South Africa, urban	Qualitative	Interviews	Opportunity sampling	- not using condoms to show love and fidelity to partner<br>- poor communication with parents<br>- non-use of contraceptives<br>- incorrect use of contraceptives<br>- unfriendly healthcare workers
**Morhe et al. [39]**	481	Ghana, urban	Quantitative	Household-based cross sectional survey	Random sampling	- being out of school<br>- marriage
**Mothiba & Maputle [30]**	100	South Africa, rural and semi-urban	Quantitative	Structured self-administered questionnaires	Simple random	- low educational expectations<br>- peer pressure<br>- family breakdown<br>- poverty

					probability sampling	- low self-esteem<br>- sexual coercion<br>- sex-based messages in the media<br>- lack of knowledge about sex/contraceptives<br>- non-use of contraceptives<br>- barriers to access of contraceptives (ex. Negative attitude of health care providers)
**Ochiogu et al. [31]**	1,280	Nigeria, urban	Quantitative	Cross sectional survey using self-administered questionnaire	Opportunity Sampling	- marital promise<br>- peer pressure<br>- sex in exchange for money<br>- family instability<br>- ignorance<br>- sexual abuse<br>- rape<br>- lack of religious commitment
**Pfeiffer et al. [40]**	750	Tanzania, urban	Quantitative	Cross-sectional survey	Cluster sampling, random sampling	- previous pregnancy<br>- cultural capital (access to media)
**Salih et al. [38]**	624	Ethiopia, urban	Quantitative	Cross-sectional questionnaire	Probability sampling, systematic random sampling	- sexually experienced friends<br>- elder sister who started sexual activity early<br>- single parent household<br>- parental divorce<br>- parents living in rural areas<br>- being born out of wedlock
